# Collecting duct carcinoma of the kidney is associated with *CDKN2A* deletion and *SLC* family gene up-regulation

**DOI:** 10.18632/oncotarget.9093

**Published:** 2016-04-28

**Authors:** Jianmin Wang, Antonios Papanicolau-Sengos, Sreenivasulu Chintala, Lei Wei, Biao Liu, Qiang Hu, Kiersten Marie Miles, Jeffrey M. Conroy, Sean T. Glenn, Manuela Costantini, Cristina Magi-Galluzzi, Sabina Signoretti, Toni Choueiri, Michele Gallucci, Steno Sentinelli, Vito M. Fazio, Maria Luana Poeta, Song Liu, Carl Morrison, Roberto Pili

**Affiliations:** ^1^ Department of Biostatistics & Bioinformatics, Roswell Park Cancer Institute, Buffalo, NY, USA; ^2^ Department of Pathology and Center for Personalized Medicine, Roswell Park Cancer Institute, Buffalo, NY, USA; ^3^ Genitourinary Program, Roswell Park Cancer Institute, Buffalo, NY, USA; ^4^ Cancer Genetics, Roswell Park Cancer Institute, Buffalo, NY, USA; ^5^ Department of Urology, Regina Elena National Cancer Institute of Rome, Rome, Italy; ^6^ Department of Pathology, Cleveland Clinic, Cleveland, OH, USA; ^7^ Department of Pathology and Kidney Cancer Program, Dana Farber, Boston, MA, USA; ^8^ Laboratory of Genetic and Clinical Pathology, University Campus BioMedico of Rome, Rome, Italy; ^9^ Department of Biosciences, Biotechnologies and Biopharmaceutics, University of Bari, Bari, Italy; ^10^ Genitourinary Program, Indiana University-Simon Cancer Center, Indianapolis, IN, USA

**Keywords:** collecting duct carcinoma, CDKN2A, solute carrier family genes

## Abstract

The genetic landscape and molecular features of collecting duct carcinoma (CDC) of the kidney remain largely unknown. Herein, we performed whole exome sequencing (WES) and transcriptome sequencing (RNASeq) on 7 CDC samples (CDC1 −7). Among the 7 samples, 4 samples with matched non-tumor tissue were used for copy number analysis by SNP array data. No recurrent somatic SNVs were observed except for *MLL*, which was found to be mutated (p.V297I and p.F407C) in 2 samples. We identified somatic SNVs in 14 other cancer census genes including: *ATM, CREBBP, PRDM1, CBFB, FBXW7, IKZF1, KDR, KRAS, NACA, NF2, NUP98, SS18, TP53*, and *ZNF521*. SNP array data identified a *CDKN2A* homozygous deletion in 3 samples and SNV analysis showed a non-sense mutation of the *CDKN2A* gene with unknown somatic status. To estimate the recurrent rate of *CDKN2A* abnormalities, we performed FISH screening of additional samples and confirmed the frequent loss (62.5%) of *CDKN2A* expression. Since cisplatin based therapy is the common treatment option for CDC, we investigated the expression of solute carrier (SLC) family transporters and found 45% alteration. In addition, *SLC7A11* (cystine transporter, xCT), a cisplatin resistance associated gene, was found to be overexpressed in 4 out of 5 (80%) cases of CDC tumors tested, as compared to matched non-tumor tissue. In summary, our study provides a comprehensive genomic analysis of CDC and identifies potential pathways suitable for targeted therapies.

## INTRODUCTION

Collecting duct renal carcinoma (CDC), also known as Bellini duct carcinoma, is a rare histological subtype (less than 2%) of renal cell carcinoma with unique clinical, histological, and pathological characteristics [1–3]. These tumors arise from the distal collecting ducts and are positioned in the renal pelvis with a gray or whitish appearance without considerable necrosis or hemorrhage [2, 3]. The clinical presentation of CDC is usually at advanced stage with symptomatic disease due to its aggressiveness, with 1-3 years disease specific survival. More than 60 percent of CDC patients manifest symptomatic disease at presentation and the common metastatic sites include lymph nodes, bone, lung, and liver [4, 5]. Epidemiological characterization of CDC in North America has revealed a prevalence of CDC in the African American male population [6, 4]. The systemic therapy offered to CDC patients is a cisplatin-based regimen, but the clinical benefit remains limited and the median survival is less than 12 months [4, 7, 8]. Treatment with targeted therapies, such as sunitinib, has also shown limited clinical benefit [9–11]. In addition, immunotherapies, including interferon alpha/gamma and interleukin, have not been shown to be significantly effective in CDC patients (*n* = 34) [5]. To determine the genetic alterations associated with chemo sensitivity, a preclinical study with human cell lines established from CDC patients revealed topoisomerase I (TOPI) expression to be associated with high *in vitro* sensitivity to TOPI and TOPII inhibitors, such as topotecan, doxorubicin, and epirubicin, suggesting TOP1 as a potential molecular target for CDC [12].

Comprehensive genomic and proteomic studies of CDC aimed at understanding the critical molecular architecture alterations associated with this tumor type have been limited. A recent report studying 17 locally advanced or metastatic CDC tumors detected 36 genomic alterations, the most common being *NF2* (29%), *SETD2* (24%), *SMARCH1* (18%), and *CDKN2A* (12%), suggesting a potential role for mTOR inhibitors in patients with *NF2* alterations [13]. A previous study of 29 CDC samples showed frequent DNA losses at 8p (9 out of 29), 16p (9 out of 29), 1p (7 out of 29), and 9p (7 out of 29); and high levels of amplifications at 13q (9 out of 29), suggesting CDC as a unique entity among kidney cancers [14].

To better understand the genomic profile of CDC tumors, we performed whole exome sequencing and RNASeq analysis of 7 CDC tumors and 4 matched non-tumor kidney tissues, as well as FISH analysis of *CDKN2A* on 16 CDC tumors. Our results revealed the frequent loss of *CDKN2A* in 62.5% (10 out of 16) and alteration of 45.3% (136 out of 300) of SLC family transporters in CDC tumors.

## RESULTS

### Genomic landscape of CDC

**Figure 1:**
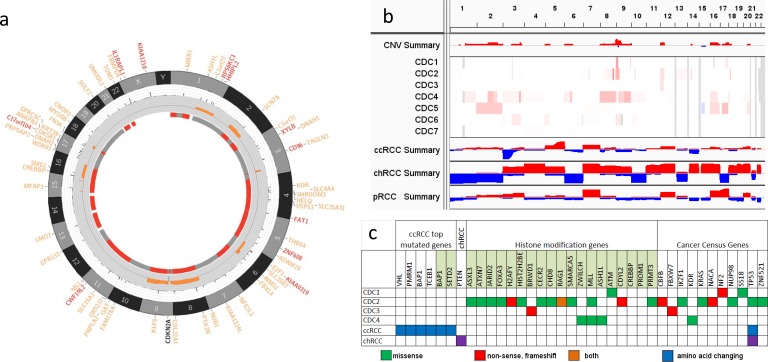
Somatic alterations in kidney CDC **a.** A representative Circos plot of CDC samples. The plot shows (from outer to inner circle) genes with somatic amino acid changes (red genes are in Cancer Census Genes), chromosomes, allele frequencies of mutations, copy number aberration (orange for gain and blue for loss), and LOH (red means LOH and grey means no LOH). **b.** Somatic CNV in 7 CDC samples and summary of CNVs in CDC, ccRCC, chRCC, and pRCC. Red color represents copy number gain and blue copy number loss. **c.** Somatic SNVs in 4 CDC samples and significantly mutated genes in ccRCC and chRCC. Green means missense mutation, red means non-sense, frameshift mutations, and orange means both. TCGA data of ccRCC and pRCC, including clinical information, somatic mutations, SNP array CNV calls, and normalized RNASeqV2, were downloaded from the TCGA data portal. Alternation status of *CDKN2A* was determined by somatic mutation calls and CNV segmentation results. If a segment overlapped with *CDKN2A* and had a logR ratio less than −0.4, *CDKN2A* was considered a loss in this sample. For gene expression data, the RSEM quantified and normalized data were first log2 transferred, followed by significant test. All statistical tests were performed using R statistical program followed by a significance test.

In our study, the overall somatic changes, including SNVs, INDELs, and CNVs, of 4 samples with matched non-tumor are shown in Figure 1. The analysis, performed as described [15–17], displays a significantly different landscape of genetic alterations in terms of somatic SNVs and INDELs (Figure 1a and Supplementary Table 1). SNP array data showed large scale somatic copy number alterations (SCNAs) and whole chromosome loss of heterozygosity (LOH) in all samples, with a small number of focal SCNAs (Supplementary Table 2). Whole exome sequencing (WES) of all 4 tumor samples identified 368 putative somatic SNVs and INDELs, including 325 missense mutations, 24 non-sense mutations, 17 frameshift indels, and 2 protein deletions.

Among the somatic mutated genes, several chromatin remodeling genes were found to be mutated, such as *PRDM1*, *CREBBP*, *MLL*, *ASXL3*, and *CHD8*. Somatic changes of epigenetic regulators represent a common theme in cancer genomes. In ccRCC studies, 3 chromatin remodeling genes, *PBRM1*, *SETD2,* and *BAP1*, are often reported as the top recurrently mutated genes [18–20]. In our study, *MLL* was the only recurrently mutated gene in 2 of the 4 samples. Fifteen somatically mutated genes in all 4 cases are in the Cancer Gene Consensus, including *TP53*, *NF2*, *KRAS*, and *IKZF.* However,** none of the top mutated genes identified in ccRCC, including *VHL*, *PBRM1*, *SETD2*, *KDM5C*, *BAP1*, *PTEN*, *MTOR,* and *PIK3CA*, were found to be altered in our study, which could be due to either the small sample size or the unique mutation spectrum of our samples. SNP array data revealed 4 samples (CDC1, CDC2, CDC4, and CDC5) as having large copy number changes and most of those were copy number gains, except for a chr15 q12 to q21.3 loss found in sample CDC5. In addition, large scale LOH was more prevalent than copy number changes (Figure 1a) and all samples, except CDC7, had LOH in multiple whole chromosomes.

### Loss of *CDKN2A/*p16 expression is common in CDC

**Figure 2:**
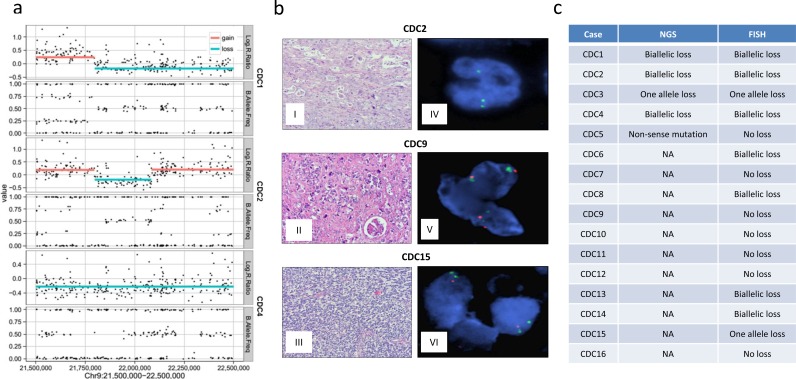
CDKN2A losses in CDC **a.** Copy number data show biallelic loss of *CDKN2A* in CDC1, CDC2, and CDC4 with negative log R ratios and normal like B allele frequencies. SNP array data log2 ratios were calculated by comparing the tumor sample signals with pooled non-tumor samples from Illumina. **b.** Representative tumor sample slides and fluorescence *in situ* hybridization (FISH) results of normal, single copy loss, and biallelic loss of *CDKN2A* loci. I, II, III: H&Es of cases CDC2, CDC9, CDC15, respectively, with infiltrating pleomorphic collecting duct carcinoma. IV, CDC2, p16 FISH with no copies each of p16 and with preserved reference probe. V, CDC9, p16 FISH with two copies each of p16 and reference probe. VI, CDC15, p16 FISH with one copy of p16 and two copies of reference probe. **c.** Tabulation of p16 loss according to NGS and FISH data. Cases CDC6-CDC16 were not sequenced by NGS, noted as NA.

Focal homozygous deletions of chr 9 p-arm in all 3 samples (CDC1, CDC2, and CDC4) included *CDKN2A*. These deletions were homozygous with corresponding copy number losses (Figure 2a). To better estimate *CDKN2A* status and to validate findings in the SNP array, we performed fluorescence *in situ* hybridization (FISH) (Figure 2b) on the 7 sequenced samples and 9 additional FFPE samples (16 total samples). The FISH results validated the homozygous losses and further identified a homozygous loss in CDC6, which was not identified by SNP array, and a heterozygous loss in CDC3. Combined with 9 FFPE samples, we found 7 samples with biallelic loss and 2 samples with single copy loss of *CDKN2A*. In CDC5, a sample without matched non-tumor, SNV analysis identified a cytosine-to-adenine transversion (c.360C > A), encoding a p.E120* non-sense mutation for *CDKN2A* gene with unknown somatic status. This sample also showed LOH at *CDKN2A* loci, suggesting that this non-sense mutation could be biallelic. Figure 2c shows the comparative analysis of NGS and FISH data on the *CDKN2A/*P16INK4a status in CDC cases. Combining the results from SNP array, exome sequencing, and FISH, we identified 8 out of 16 (50%) homozygous losses and 2 heterozygous losses (12.5%) in all samples examined. A recent study showed similar results with genomic alteration of *CDKN2A* in 12% (2 out of 17) of CDC cases, one with homozygous gene deletion and another with truncation [13]. The *CDKN2A* gene encodes several proteins, including p16 (INK4a) and p14 (ARF), which are tumor suppressor genes that regulate the cell cycle and protect p53 [21, 22]. Loss of *CDKN2A* expression has been associated** with the induction of *CDK4/CDK6*. Selective inhibitors for *CDK4/CDK6* are currently in clinical development and, based on our findings, may represent a rational therapeutic strategy for CDC.

**Figure 3:**
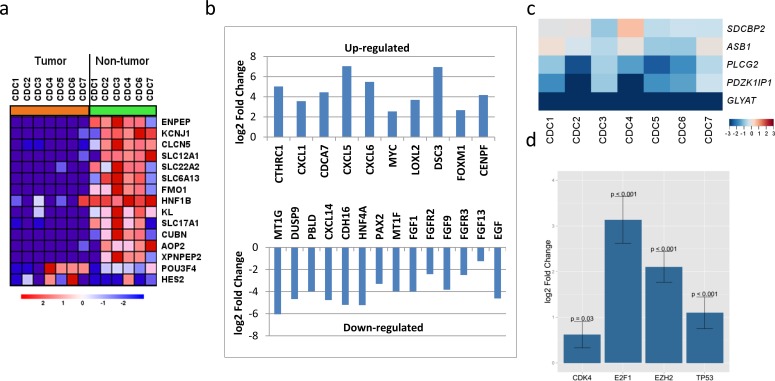
RNASeq profiles of CDC gene expression **a.** Kidney specific genes were down-regulated in all CDC tumor samples. **b.** Selected top up- and down-regulated genes that have been associated with cancer prognosis. **c.** Gene expression changes (log2 fold changes) of a five-gene signature defined by a study of genomic alterations in non-clear cell RCC (http://www.nature.com/ng/journal/v47/n1/full/ng.3146.html) to classify non-ccRCCs. CDC shows a distinct pattern for those five genes compared with three subtypes of non-ccRCC. **d.** Four down-steam genes (*CDK4*, *E2F1*, *EZH2*, and *TP53*) of *CDKN2A* were all found to be significantly up-regulated by RNASeq analysis.

The paired analysis of RNASeq data revealed 2879 up-regulated and 1951 down-regulated genes in tumor samples, as compared with matched non-tumor samples. The principal component analysis (PCA) showed the separation of tumor and matched non-tumor samples at the first component and one tumor sample (CDC4) with a distinct expression profile at the second component. This was also seen in un-supervised clustering analysis. MYC amplification was identified in 1 sample (CDC2) and up-regulation in all 4 tumor samples analyzed (adjusted *p*-value < 0.001) (Figure 3b). The cell division cycle associated 7 (*CDCA7*) gene, one of the direct MYC targets, was also found to be up-regulated (Figure 3b). High-mobility group AT-hook 2 (*HMGA2*) and Collagen Triple Helix Repeat Containing 1 (*CTHRC1*), the top two up-regulated genes (according to our data) with log2 fold changes greater than 6, have been reported in the literature to be overly expressed in several cancer types and to be related to tumor prognosis [23–26]. A review of TCGA data showed *HMGA2* to be overexpressed in ccRCC, but not in pRCC (Figure 7) and survival analysis showed significant poor outcome for the high *HGMA2* expression group in both ccRCC and pRCC (log-rank test, *p*-value < 0.001 and 0.004, and adjusted *p*-value of < 0.001 and 0.026 for ccRCC and pRCC, respectively). Similarly, *CTHRC1* was found to be significantly up-regulated in pRCC, but not in ccRCC, and the survival analysis showed significant poor overall survival for the high *CTHRC1* expression group (log-rank test, *p*-value = 0.013 and 0.001, and adjusted *p*-value 0.053 and < 0.001 for ccRCC and pRCC, respectively). To support the significance of *CDKN2A* loss in renal cancer, we further analyzed the available TCGA data and found worst survival for ccRCC and pRCC patients with *CDKN2A* alteration, as compared to wild type (Figure 8). Based on these observations, up-regulation of *HMGA2* and *CTHRC1*, together with loss of *CDKN2A*, may serve as prognosis markers in renal cell carcinoma.

### Alteration of the solute carrier (SLC) family members

**Figure 4:**
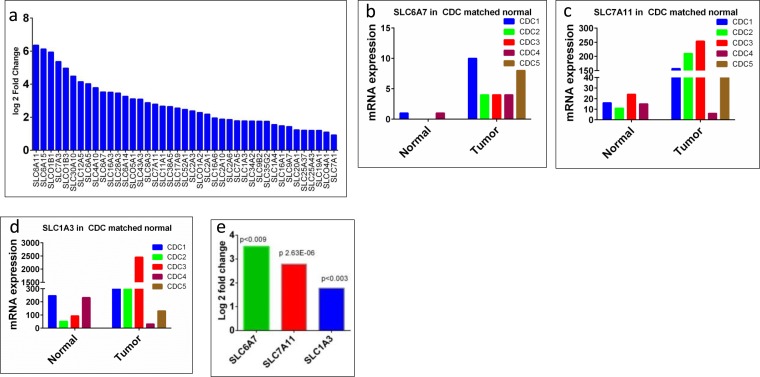
*SLC* family genes upregulated in CDC **a.** *SLC* family genes overexpressed in CDC tumors showed as log2 fold change. All the genes overexpressed were significantly different in CDC tumors, compared to matched non-tumor kidney. **b.** *SLC6A7* mRNA expression in 4 matched non-tumor kidney and 5 CDC (1 non-matched) tumors. **c.** *SLC7A11* mRNA expression in 4 matched non-tumor kidney and 5 CDC (1 non-matched) tumors. **d.** *SLC1A3* mRNA expression in 4 matched normal kidney and 5 CDC (1 non-matched) tumors. **e.** Significant overexpression (log2 fold change) of drug resistance genes *SLC6A7*, *SLC7A11,* and *SLC1A3* in CDC tumors.

Since collecting ducts of the kidney are critical for transport, reabsorption, and excretion of several important electrolytes, we were interested in assessing the status of the membrane transporters, in particular, the solute carrier (SLC) family members, which play a critical role in transportation and absorption of electrolytes and drug resistance. Our RNASeq data analysis found 136 *SLC* family genes to be altered (41 up-regulated and 95 down-regulated; Figure 4a and Figure 9), which is 3.8 % (136 out of 4,830) of the total gene alterations and 45.3% (136 out of 300) of the *SLC* group of family members [27, 28], suggesting a potential role in CDC. The function of these altered transporters range from transport of amino acid, carbohydrates, metals, vitamins, and nucleotide sugars [27, 28]. The top 5 up-regulated genes (log2 fold change of 6.4 to 5.0) were *SLC6A11, SLC6A15, SLCO1B1, SLC7A3,* and *SLCO1B3* (Figure 4a), which are involved in the transport of gamma-aminobutyric acid (GABA), neutral amino acids (leucine, valine and methionine), organic anion update such as methotrexate, prostaglandin E2, cationic amino acids-arginine, lysine and ornithine, and organic anion methotrexate, respectively (Supplementary Table 3). The top down-regulated genes (log2 fold change of −8.4 to 7.6) were *SLC22A12, SLC5A12, SLC47A2, SLC22A6,* and *SLC12A1* (Figure 9, Supplementary Table 4).

**Figure 5:**
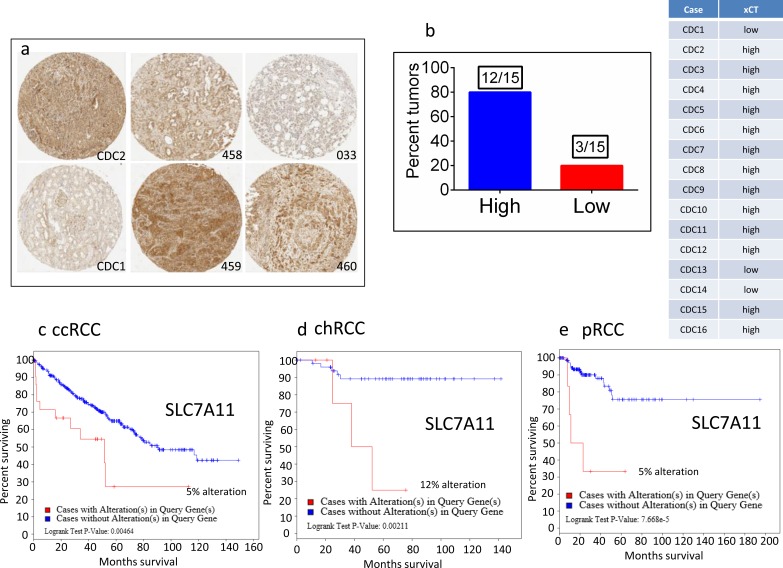
Overexpression of xCT in CDC tumors and the association of xCT (SLC7A11) overexpression with overall poor survival in ccRCC, chRCC and pRCC patients **a.**. Immunohistochemical detection of xCT was performed on CDC tumors using xCT antibody (5ug/ml, Abcam, MA). The numbers denoted in the figures are de-identified numbers of CDC tumors in the TMA. CDC1 and CDC2 tumors are included in the genomic profiling studies. Photmicrographs were captured using the Aperio Webscope Spectrum. **b.** Percent tumors (80%, 12 out of 15) express high levels of xCT. TCGA data analysis revealed the significant poor survival of RCC patients with xCT upregulation: **c.** ccRCC patients, Logrank Test P-value 0.00464; **d.** chRCC patients, Logrank Test P-value:0.00211, **e.** pRCC patients, Logrank Test P-value: 7.668e-5.

**Figure 6:**
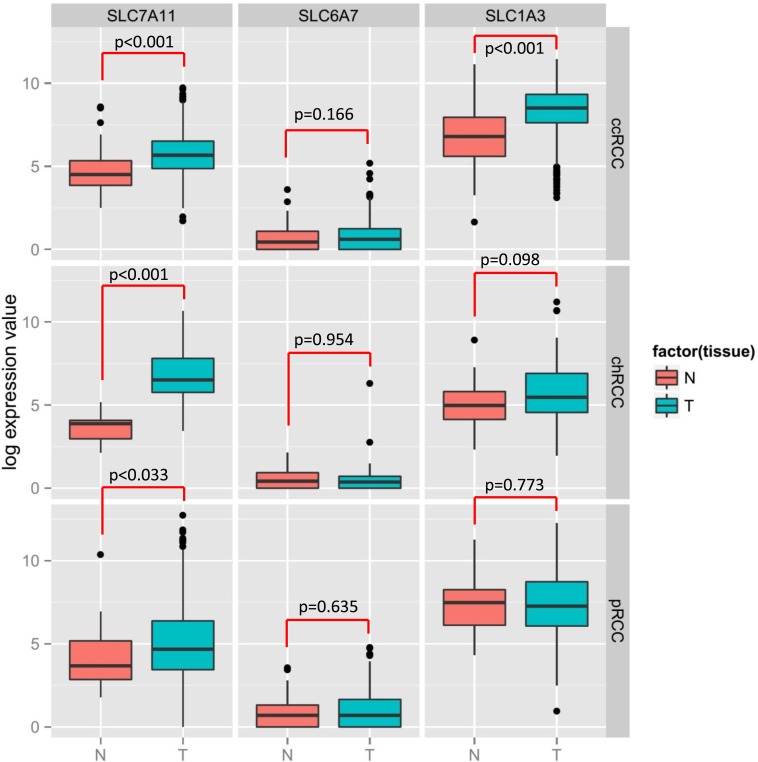
Significant upregulation of SLC7A11 in 3 subtypes of RCC (ccRCC, chRCC and pRCC) tumors compared to normal tissue TCGA data analysis revealed the differential expression of SLC7A11, SLC6A7 and SLC1A3 among the 3 subtypes of RCC tumors. Left panel showing the SLC7A11 expression in ccRCC, chRCC and pRCC; middle panel with SLC6A7, right panel with SLC1A3. *N* = Normal tissue, T = Tumor tissue

Thus, the critical analysis of individual amino acid transporters of *SLC* family genes from the up-regulated group led us to investigate three transporters: *SLC7A11* (xCT, cystine transporter), *SLC1A3* (GLAST, glutamate and aspartate transporter), and *SLC6A7* (PROT, proline transporter) (Figure 4), which are known drug resistance markers [28–37]. Recent studies have reported xCT (SLC7A11), in particular, as a drug resistance maker and a novel target for therapeutic interventions in lung, breast, head & neck, and bladder cancers [35, 38–43]. Selective targeting of the glutamate and aspartate transporter, GLAST, has shown to reverse drug resistance in colorectal, ovarian, and breast cancer models [32, 44–46]. Recently, the proline transporter, PROT, was reported to have a significant role in the tumor microenvironment [29] and limiting of intracellular proline inhibits proliferation of renal cancer cells [47]. In accordance, our RNA sequence data showed the up-regulation of *SLC7A11* (Figure 4c) and *SLC1A3* (Figure 4d) mRNA in 80% of the cases (4 out of 5), with *SLC6A7* (Figure 4b) up-regulated in 100% of the cases. In addition, immunohistochemical analysis of CDC tumors (*n* = 15) revealed the overexpression of xCT (*SLC7A11)* in 80% (12 out of 15) of the cases (Figure 5a, 5b). Taken together, our results demonstrate, for the first time, that overexpression of xCT, a cisplatin resistance associated marker, in CDC tumors, and suggest that xCT targeted combination therapies may be beneficial to CDC patients. In order to evaluate the *SLC7A11* in other subtypes of kidney cancer, we have used the TCGA data [48, 49] and found that *SLC7A11* significant upregulation in RCC subtypes ccRCC, chromophobe RCC (chRCC) and papillary RCC (pRCC) (Figure 6) and the upregulation was associated with significant poor survival of patients (Figure 5c, 5d, 5e). The other two transporters (*SLC6A7* and *SLC1A3*) are not significantly upregulated (except *SLC1A3* in ccRCC) in ccRCC, chRCC and pRCC and their association with the overall survival of RCC patients is not significant (Figure 6 and 11).

**Figure 7:**
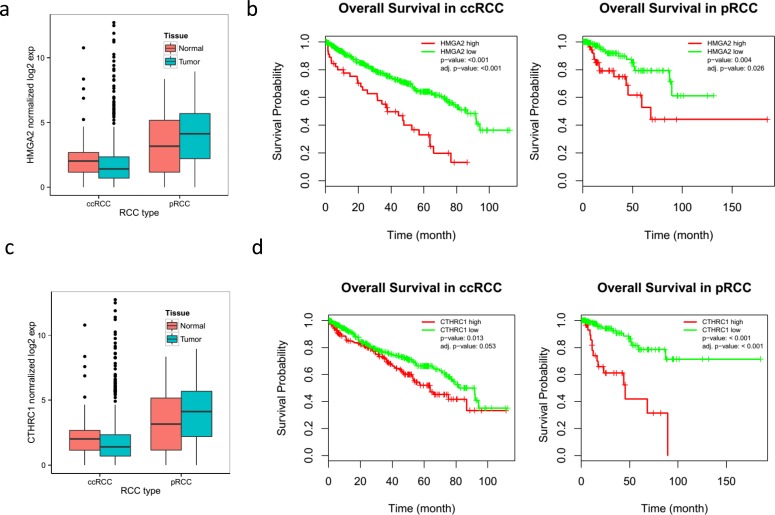
Overall poor survival in RCC patients with overexpression of *HMGA2* and *CTHRC1* which were found highly upregulated (top two genes) in CDC tumors The available TCGA data were downloaded and utilized to determine the survival probability in ccRCC patients in order to determine the significance of overexpression of these genes in CDC tumors. a. *HMGA2* expression in ccRCC and pRCC. b. Overall survival probability of ccRCC and pRCC patients with high expression of *HMGA2*. c. *CTHRC1* expression in ccRCC and pRCC. d. Overall survival probability of ccRCC and pRCC patients with high expression of *CTHRC1*.

**Figure 8:**
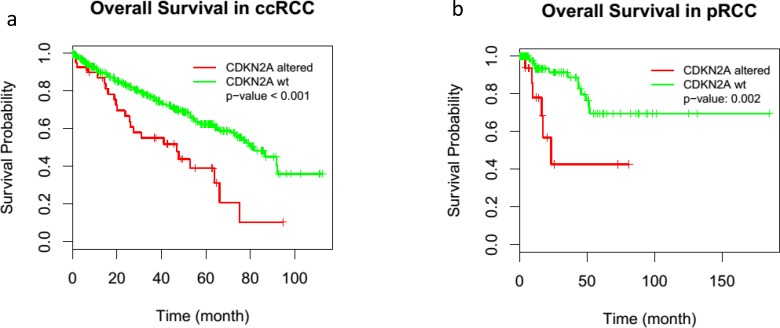
Alteration of *CDKN2A* and renal cancer patients survival *CDKN2A*/p16INK4A alteration significantly decreased the survival in ccRCC **a.** and pRCC patients **b.**. TCGA data were used to evaluate the survival probability in renal cancer patients.

## DISCUSSION

Collecting renal cell carcinoma is a rare subtype of kidney cancer, aggressive in nature and in general resistant to chemotherapy and targeted therapies. One important limitation in the understanding the biology of CDC is the lack of relevant preclinical models. Clinical studies have proposed cisplatin based chemotherapy as standard of care for CDC. Further, triple combination of bevacizumab, gemcitabine and cisplatin/carboplatin has been reported to be effective in terms of progression-free survival and overall survival as compared to platinum-based chemotherapy [50]. Additionally, double HER2 blockade has been shown to have activity in disseminated CDC [51]. In the majority of CDC cases, surgical treatment did not result in cure [52]. A case report study on metastatic CDC revealed a favorable response to multikinase inhibitor sunitinib [53] suggested the role of targeted therapy in CDC. Several studies have provided genomic profiling and molecular targeted therapies for the most common types of kidney cancer, such as clear cell renal cell carcinoma, but very limited studies have been focused on CDC. A recent genomic profiling study [13] has suggested a potential therapeutic role for mTOR inhibitors in CDC with NF2 alterations. Additional studies are urgently needed to better understand the CDC molecular signature and to develop novel therapeutic agents and effective combination therapies.

Since we observed that the *CDKN2A* was the most frequently altered gene in our CD samples, we assessed the p16 interacting proteins using the STRING10 biological database, a network which shows the known and predicted protein interactions. Our data suggest that p16 interacts with known oncogenic pathways CDK4, TP53, MYC, and MDM2 (Figure 10), and may play a biological role in CDC. Our RNASeq analysis confirmed the overexpression of CDK4, TP53, and MYC** in the tumor samples, suggesting that indeed p16 deletion may play a critical role in the biology of CDC tumors by the overexpression of oncogenic signaling pathways. In particular, CDK4 upregulation in CDC tumors provides a potential target for therapeutic intervention since selective CDK4/CK6 inhibitors are now available for clinical testing.

**Figure 9:**
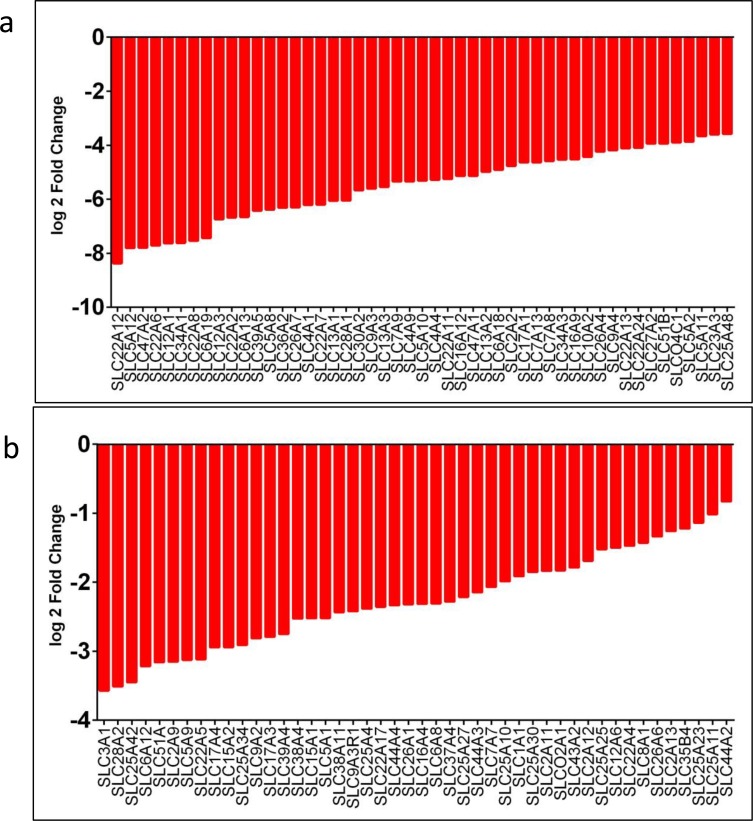
SLC family genes downregulated in CDC tumors compared to non-tumor kidney SLC family genes downregulated in CDC tumors showed as log2 fold changes from 8.369 to 3.580 **a.** and 3.569 to 0.827 **b.**. All the genes listed are downregulated significantly in CDC tumors compared to matched non-tumor kidney. RNASeq data was used to identify *SLC* family gene expression levels in CDC tumors. The difference in the expression levels as a log2 fold change in expression levels was observed.

**Figure 10:**
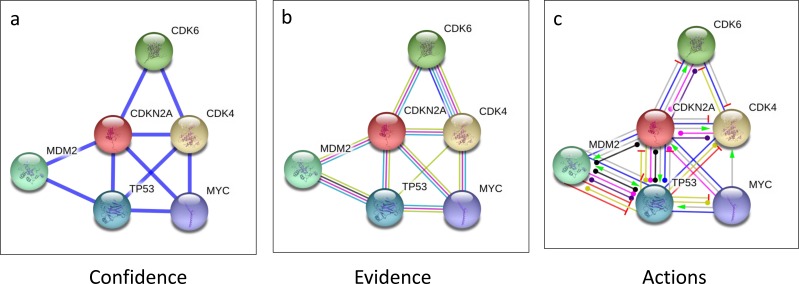
CDKN2A interacting proteins To determine human *CDKN2A* interacting proteins, STRING10 (http://string-db.org/) database which provides known and predicted protein interactions was used. Interaction views of confidence **a.**, evidence **b.** and actions **c.** are shown using parameters of highest confidence (0.900) and no more than 5 interactions. Protein interaction data show that *CDKN2A* interacts with CDK4 and TP53 (listed top 5), E2F1 and EZH2, (listed top 20, data not shown), which were found overexpressed by RNASeq analysis (Figure 3d), suggesting the functional significance of *CDKN2A* deletion in CDC tumors.

In our study we have also observed several SLC family genes upregulation including *SLC7A11* (xCT) a marker associated with cisplatin resistance [34, 35, 37, 54]. To confirm the RNASeq data, we have evaluated the xCT expression in CDC tumors and found overexpression in 80% of cases (Figure 5a, 5b). Based on this observation we can speculate that upregulation of xCT may be responsible for the lack of CDC sensitivity to cisplatin, and targeted therapies aims to inhibit xCT may have a therapeutic benefit. In addition, we found upregulation of several genes such as *HMGA2*, *CTHRC1* (top two genes) whose role in cancer progression and survival is well established (Figure 7). Recently, *HMGA2* overexpression has been shown associated with cisplatin resistance in human non-small clear lung cancer [55] supports our findings to indicate that overexpressed genes contribute cisplatin based therapy resistance in CDC. Furthermore, its overexpression has been reported to associate with gemcitabine resistance in pancreatic cancer [56], sunitinib in hepatocellular carcinoma [57]. Overexpression of *CTHRC1* that encodes for a secretary protein involved in vascular remodeling through limiting collagen matrix deposition, has been associated with pancreatic cancer cells migration and metastasis [58], and melanoma invasiveness [59].

In summary, our study provides a comprehensive genomic analysis of CDC and identified the loss of *CDKN2A* expression and dysregulation of several transporters as frequent molecular alterations in this disease. We recognize the limited sample size of our report due to the rarity of CDC. Despite this limitation, we believe that our observations provide important findings which have a potential translational impact on the understanding of the well-known CDC chemotherapy resistance. These findings have immediate translational implications by suggesting rational combination strategies that may improve the clinical outcome for CDC patients.

**Figure 11:**
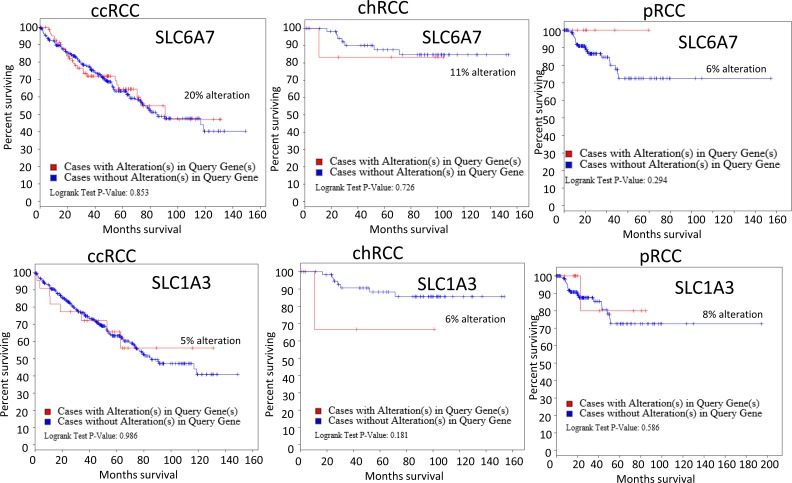
Alteration of *SLC6A7* and *SLC1A3* association with RCC patients survival Upper panel-TCGA data analysis of *SLC6A7* alteration in 3 subtypes of RCC (ccRCC, chRCC and pRCC). Lower panel- *SLC1A3* alteration and RCC patients’ survival.

## MATERIALS AND METHODS

### Patient samples

Frozen patient tumor samples and matched normal kidney tissues were obtained from the tissue bank at the participating institutions. These tissues were originally collected and processed according of the Institutional Review Board at each institution and were received at Roswell Park Cancer Institute as de-identified samples.

### Exome sequencing read mapping and detection of somatic mutations

High quality paired-end reads passing Illumina RTA filter were aligned to the NCBI human reference genome (hg19) using BWA ([16]). PCR duplicated reads are marked and removed using Picard (http://picard.sourceforge.net/). Putative SNVs and indels were identified by running variation detection module of Bambino [15]. A putative mutation was further filtered based on the following criteria: (1) the alternative allele is absent in the paired non-tumor sample; (2) Fisher exact test p-value shows that number of reads with non-reference allele is significant higher in tumor sample; (3) mutant allele is present in both orientation; (4) absence of photopolymers at variant position. For putative indels, the filter process re-aligns all reads in both tumor and paired normal at the indel site with a template sequence generated by replacing reference allele with mutant one. After these two steps, germline variants are effectively removed. The putative variants were annotated using ANNOVAR [17] using NCBI RefSeq database.

### Copy number analysis

Sample pairs (*n* = 5) with SNP array data, log2 ratios were calculated by comparing the tumor sample signals with pooled normal samples from Illumina. Then OncoSNP R package [60] was used to perform segmentation analysis on generating LOH and CNV segments. The segmentation results were further manually reviewed to identify missing focal copy number changes.

### TCGA data analysis

TCGA data for ccRCC and pRCC including clinical information, somatic mutations, SNP array CNV calls, and normalized RNASeqV2 are downloaded from TCGA data portal. Alternation status of *CDKN2A* is determined by somatic mutation calls and CNV segmentation results. If a segment overlaps with *CDKN2A* has logR ratio less than −0.4, *CDKN2A* is considered loss in this sample. For gene expression data, the RSEM quantified and normalized data are first log2 transferred followed by significant test. All statistical tests are performed using R statistical program.

### RNASeq data analysis

Raw reads passed quality filter was first pre-processed using tools FASTQC for quality control followed by sequence mapping using TopHat to the human reference genome and ENSEMBLE annotation database. HTSeq [61] was used to count number of reads mapped to each gene and transcript. Differentially expressed genes were identified using DESeq2 [62], a variance-analysis package developed to infer the statically significant difference in RNA-seq data. Multiple testing corrections will be performed in DESeq2. The list of differentially expressed genes (DEGs) was analyzed for enriched pathway analysis using GSAA [63].

### Immunohistochemical analysis

Immunohistochemical detection of xCT (encoded by SLC7A11 gene) in CDC tumors arranged in tissue microarray (TMA) available in Roswell Park Cancer Institute pathology core (RPCI_GUCa15) were used for detection of xCT. Triplicates tumor sections of each CDC tumor cores (1.0mm), de-identified numbers (CDC2, 458, 033, CDC1, 459 and 460) in TMA were utilized. CDC1 and CDC2 tumors were included in the current genomic profile studies. Additional cohort of 15 CDC tumors obtained from outside institute was also evaluated for the xCT expression. Rabbit polyclonal xCT antibody (Abcam, MA) was used at the dilution of 5 μg/ml to evaluate xCT using the protocol described [64]. Briefly, paraffin-embedded TMA sections were cut in 5 uM thickness sections, deparaffinized, rehydrated and subjected to heat mediated antigen unmasking in 10mM sodium citrate buffer (pH6.0). Quenching of endogenous peroxidases was done with 3% Hydrogen peroxide. Blocking was done with 1% bovine serum albumin in phosphate buffered saline for 1h followed by incubation with xCT primary antibody overnight. Sections were incubated with horseradish conjugated anti-Rabbit secondary antibody (Vector Laboratories, Burlington, CA) for 1hour and developed in diaminobezidine (Dako, Carpinteria, CA) and hematoxylin counterstain. Immunoscoring was performed by determining the intensity of staining level as low (2-20% positive cells) and high (80-100% positive cells).

### Fluorescence *in situ* hybridization (FISH)

To verify the *CDKN2A* NGS findings, we performed fluorescence *in situ* hybridization (FISH) on five sequenced CDC cases, followed by eleven additional cases obtained from outside institutions. *CDKN2A* enumeration by FISH was done using the commercially available combined *CDKN2A/CEP9* probes (Vysis, Downers Grove, IL). Two hundred neoplastic cells were evaluated with the *CDKN2A/CEP9* probe set for each of the collecting duct carcinoma cases. For each case, ratios were obtained by dividing the average number of *CDKN2A* probes per cell by the average number of CEP9 probes per cell. A ratio above 0.75 was considered “no loss of *CDKN2A*”. A ratio between 0.5 and 0.75 was considered a “heterozygous loss of *CDKN2A*”. A ratio below 0.5 was considered a “biallelic loss of *CDKN2A*”.

### SUPPLEMENTARY MATERIALS TABLES








